# An Overview of Whole Grain Regulations, Recommendations and Research across Southeast Asia

**DOI:** 10.3390/nu10060752

**Published:** 2018-06-11

**Authors:** Iain A Brownlee, Ece Durukan, Gabriel Masset, Sinead Hopkins, E-Siong Tee

**Affiliations:** 1 Newcastle Research and Innovation Institute, Devan Nair Building, Singapore 600201, Singapore; 2 CSIRO Nutrition & Health Program, SAHMRI Building, North Terrace, Adelaide, SA 5000, Australia; 3 Cereal Partners Worldwide, Dubai 17327, UAE; Ece.Durukan@AE.nestle.com; 4 Cereal Partners Worldwide, CH-1350 Orbe, Switzerland; Gabriel.masset@rd.nestle.com (G.M.); Sinead.Hopkins@rd.nestle.com (S.H.); 5 TES Nutrihealth Strategic Consultancy, 46, Jalan SS22/32, Petaling Jaya 47400, Selangor, Malaysia; estee@nutrihealth.com.my

**Keywords:** whole grains, wholegrain foods, ASEAN, ASEAN harmonization, food-based dietary guidelines

## Abstract

The Association of Southeast Asian Nations (ASEAN) is a diverse region that is experiencing economic growth and increased non-communicable disease burden. This paper aims to evaluate the current regulations, dietary recommendations and research related to whole grains in this region. To do this, a systematic literature review was carried out and information was collected on regulations and dietary recommendations from each member state. The majority of publications on whole grains from the region (99 of 147) were in the area of food science and technology, with few observational studies (*n* = 13) and human intervention studies (*n* = 10) related to whole grains being apparent. Information from six countries (Indonesia, Malaysia, The Philippines, Singapore, Thailand and Vietnam) was available. Wholegrain food-labelling regulations were only noted in Malaysia and Singapore. Public health recommendation related to whole grains were apparent in four countries (Indonesia, Malaysia, The Philippines, Singapore), while recent intake data from whole grains was only apparent from Malaysia, The Philippines and Singapore. In all cases, consumption of whole grains appeared to be very low. These findings highlight a need for further monitoring of dietary intake in the region and further strategies targeted at increasing the intake of whole grains.

## 1. Introduction

Southeast Asia is a region that produces high amounts of key food commodities and includes areas of divergent socio-economic status. The major grain crops produced in the region are rice and maize [1]. The region experiences public health challenges with undernutrition, as well as over-nutrition [2,3]. In particular, the prevalence of obesity [4] and type II diabetes [5] have dramatically increased within some countries in this region recently. Therefore, a major future public health focus in Southeast Asia will be reducing the prevalence of non-communicable diseases.

Consistent observational evidence suggests that intake of wholegrain foods is part of an ideal dietary template that is associated with reducing the risk of premature, mortality, cardiovascular disease, metabolic health issues like type II diabetes and colorectal (as well as other site) cancers [6,7,8] and the rate of weight gain over the life-course [9,10]. However, dietary intake of whole grains tends to be lower than recommended levels in many parts of the world [11,12,13,14]. The aforementioned evidence base on whole grain intake and non-communicable disease risk has largely been dominated by studies carried out in Europe and North America, although it appears that studies from other regions of the world are also starting to add to a global body of evidence [15]. The burden of non-communicable diseases is enormous worldwide. In comparison to worldwide trends, the estimated rate of disability-adjusted life years lost linked to low whole grain intake in Southeast Asia, East Asia and Oceania appears to be increasing rapidly (over 3 million additional years lost between 2005 and 2016) [16], yet country-specific, objective intake data in Southeast Asia are limited.

The Association of Southeast Asian Nations (ASEAN) consists of ten member states (Brunei Darussalam, Cambodia, Indonesia, Lao People’s Democratic Republic, Malaysia, Myanmar, The Philippines, Singapore, Thailand and Vietnam) and is currently moving towards becoming a free trade region [17] and has strong economic ties with China [18]. This will likely affect all intra-ASEAN and external trade in the forthcoming years. Food-related regulations between countries are likely to impact on the future of food provision in the region and, in the case of whole grains, could impact on public health. The regulations and health-related recommendations pertaining to whole grains/wholegrain foods and the current patterns of their consumption across Southeast Asia have not, to the authors’ knowledge, previously been considered in detail. Therefore, the purpose of this paper was to provide an analysis of the current state of play of regulations, dietary recommendations and research related to whole grains in/across ASEAN countries. In order to achieve this, a systematic literature search was carried out and information was collected on health claims and regulations from each member state via survey.

## 2. Methods

### 2.1. Literature Search

**Figure 1:**
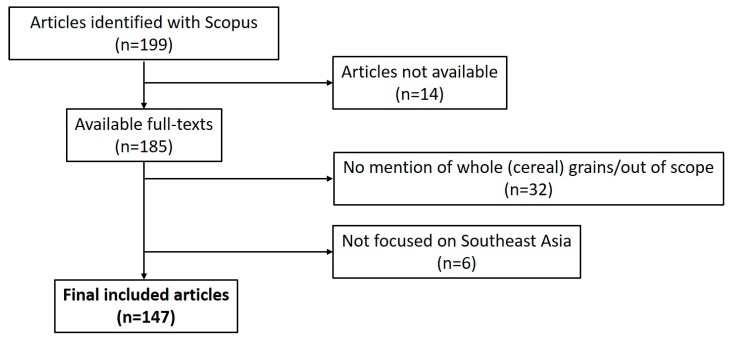
Overview of the literature search approach.

An exhaustive literature search was carried out by a single researcher (IAB) using the Scopus^®^ database using search terms specific to each ASEAN nationality and country (e.g., “Thai” and “Thailand” or Singapor*) and a wide range of whole grain-related terms (e.g., “whole grain”, “wholegrain”, “brown rice”, “red rice”, “wholemeal”, “whole wheat”, “barley” and “oats”). See Table S1 for a full list of search terms. Included articles were broadly categorised into one of four major areas of food and nutrition-related research (food production (by agriculture or other means), food science and technology, consumer sciences, and nutrition). Articles that involved grain, participants or research institutes from ASEAN were included within the final results. A summary of the literature review is included in Figure 1. The search was carried out on the week of the 22 November 2017.

### 2.2. Collection of Data on Whole Grain Regulations and Recommendations

A 21-question template (see headings on Tables 2–5) was developed in order to collect information on whole grain dietary recommendations, regulations and wholegrain food consumption and was completed with support from in-country nutritionists (by email) within the authors’ network who had a strong background in whole grains. Qualitative and categorical information was subsequently anonymized and collated by one researcher (IAB). The authors checked the information provided wherever possible. Information was collected between the 17 October 2017 and the 1 March 2018.

## 3. Results

### 3.1. Literature Review

There were a total of 147 studies noted that met the inclusion criteria (see Figure 1). A full list of these studies is available in Table S1. As highlighted in Table 1, the majority of these studies were in the area of food science and technology (*n* = 99), with a large number of these studies relating to food science. In particular, a number of these previous studies have focused on the nutrient or phytochemical content of whole grains (*n* = 43 in both cases) with a reasonable number of studies also evaluating safety aspects of whole grains (*n* = 9), particularly in relation to mycological safety assessment (*n* = 6). Thailand (*n* = 80) and Malaysia (*n* = 33) were the countries with the highest number of research articles on whole grains. Only one article (which included data from multiple countries) related to whole grains in Lao People’s Democratic Republic [19], while there were no articles that related to whole grains and Brunei Darussalam or Myanmar.

Animal or in vitro experimental studies, population-based observational studies and acute or long-term human intervention studies were all included under the umbrella term of “nutrition” research. Of the included 33 studies in this area, there was a reasonably even split across human intervention studies, observational studies and experimental studies (*n* = 10, 13 and 10 respectively). Three of the included observational studies were based on the dietary habit of Southeast Asian migrants who were currently residing in the US [20,21,22]. A number of these studies had assessed wholegrain intake in specific populations groups, such as diabetes patients [23] and cancer patients [24,25], smokers [26], Singaporeans of Chinese ethnic origin [27], pregnant women [28] and fast food consumers [29]. One study evaluated the broad dietary patterns of Thai adults but did not present specific data on wholegrain food consumption [30]. The remaining two observational studies [31,32] were representative of dietary intake at a national level (in children) [31,32] and were also highlighted within the information collection whole grain recommendations, regulations and consumption from member states (see below).

Half of the consumer studies found in the literature search related to sensory analysis of whole grains or products [33,34,35] containing whole grains [36,37], while other studies had evaluated consumers’ attitudes, acceptance and knowledge of wholegrain foods [38,39,40,41]. The final study had presented an overview of public health strategies aimed at increasing consumption of whole grains in children [42].

Most of the human intervention studies were acute in nature, with 5 studies related to assessment of postprandial glycaemic response [37,43,44,45,46], one on satiety [47] and two on iron absorption from single meals containing whole grains [48,49]. Two of these studies did not draw comparisons between the acute physiological impacts of rice consumption to non-whole grain comparators [37,49]. Only one of the five studies that compared glycaemic response of wholegrain foods to refined grain comparators studies suggested that whole grains blunted the postprandial glucose response [49]. One study [43] focused on (commercially-available, whole, multi-grain and refined grain) breads while the others compared whole and refined rice.

Two of the intervention studies were longer-term randomised, controlled trials that suggested a benefit of consuming whole grains to markers of health versus refined grain comparators. In one of these, a 4-week intervention where white rice was replaced for oats resulted in significant reductions in total and LDL cholesterol in hyperlipidaemic Thai adults [50]. In the other, pre-germinated brown rice significantly improved anthropometric measures and lipidaemic and glycaemic markers in Vietnamese women with impaired glycaemic control [51].

### 3.2. Data on Whole Grain Recommendations, Regulations and Consumption

Of the ten ASEAN member states, responses were received from six in-country nutritionists (from Indonesia, Malaysia, The Philippines, Singapore, Thailand and Vietnam) after follow-up. These findings are outlined below in Table 2, Table 3, Table 4 and Table 5. The authors could not find any information to support the presence of recommendations, regulations or intake data related to whole grains from the four non-respondent countries (Brunei Darussalam, Cambodia, Lao People’s Democratic Republic and Myanmar). Table 2 highlights that four countries in the region included suggestions for whole grain intake within their food-based public health guidelines. In Indonesia, the Nutrition Balance Guidelines note that “whole grains such as corn, red rice, black sticky rice… are high in fibre”. In the Malaysian Dietary Guidelines 2010, Key message 4 implicitly states that whole grains are the preferred type of starchy food that should be consumed among “rice, other cereal products and tubers”. The “Pinggang Pinoy” (which translates to “Healthy Food Plate for Filipino Adults) emphasizes that whole grains are the preferred food group amongst energy-providing food items. In Singapore, the category of “Rice and Alternatives” has recently been updated to “Whole Grains and Alternatives” in a bid to underline the importance of wholegrain food intake. The latter three countries provide additional detail within the national food-based dietary guidelines on mounts of whole grains to consume and to explain their association with positive health outcomes, particularly reduced risk of cardiovascular disease. The public health agencies of 2 countries (The Philippines and Singapore) appeared to have educational or engagement materials aimed at increasing intake of whole grains at a national level.

In comparison to public health agencies, other in-country professional bodies tended to be less frequently involved in making suggestions for whole grain intake (see Table 3). The only countries that there was evidence of this occurring were Malaysia and Singapore, where recommendations (from the Nutrition Society of Malaysia and Singapore Nutrition and Dietetic Association respectively) had been drafted in partnership with or with clear reference to the national public health agencies.

Only two of the six respondent countries had regulatory frameworks governing wholegrain foods definitions or whole grain-related claims (see Table 4). In both Indonesia and Singapore, there was a requirement to include the percentage of whole grains in a product labelled “Whole Grain”, although the types of grain did not have to be stated. In Indonesia, foods that are “whole, broken, or flaked grain, including rice” and “breakfast cereals, including rolled oats” are the product types that can bear claims to being wholegrain foods, with the latter requiring a minimum content of 25% whole grains. In Singapore, products containing whole grains that meet specific requirement for nutrient profiles (i.e., that meet requirements for low-fat/high fibre content claims) can make broad claims in relation to healthy diets rich in whole grains, fruits and vegetables reducing the risk of major non-communicable diseases, although there is no stated minimum percentage of whole grains that should be included. However, products must state the percentage of whole grains that they contain on the pack [65,66]. Although Malaysia does not currently have regulations in place in this area, a draft of proposed updates to food regulations that would include this is currently open for public consultation [67]. In other countries (Vietnam and Thailand), it was somewhat uncertain whether the lack of whole grain regulations in other ASEAN countries would mean that claims (whether accurate or not) could be made.

Table 5 highlights the availability of observational data on whole grain consumption habits across the ASEAN region. Only 3 respondent countries (Malaysia, Singapore and The Philippines) had any recent estimates of wholegrain food intake, with longitudinal data on wholegrain food intake only being available for adults (18–69-year-olds) in Singapore. While Thailand did not have recent consumption data per se, some national data on intake of food items, including some wholegrain foods like brown rice from 2002–2005 exist [72].

For the three countries where data on intake were available, it appeared that wholegrain foods were consumed infrequently. Children’s dietary intake data in Malaysia and Singapore had been expressed in grams per day (mean of 2.3 g/day (standard deviation 5.8 g/day) and median of 0·00 (interquartile range of 0·00–9·39 across the respective samples), with a slightly higher proportion of children in Singapore consuming whole grains during the data collection period (38.3%) compared to Malaysia (24.9%). A low proportion of Filipino children (1 in 20) and adults (1 in 10) consumed whole grains [73], with maize grits and other maize products being the most commonly consumed foods in this survey defined as being whole grain.

## 4. Discussion

It appears that the previously published research on whole grains in Southeast Asia has provided a considerable body of literature in relation to food science and technology but is perhaps somewhat more limited in consumer- and health-related studies. A lack of evidence from observational studies and randomized, controlled trials in Southeast Asia has the potential to limit the tailoring of “ideal” dietary patterns to region- or country-specific dietary intakes. Filling this evidence gap on whole and refined grain intake and health in Southeast Asia will avoid making potentially inappropriate inferences from data collected elsewhere in the world. Better understanding of the consumer base in the region is also likely to benefit public health messaging around increasing intake of whole grains at a population level and should also help in the development of innovative wholegrain food products.

Consideration of the proportion of articles within each research area at least suggests the available expertise in the region. Over 60% of the studies published to date have come from research institutes in Thailand and Malaysia. Despite ASEAN member states being major exporters of grains, particularly rice [76], relatively few studies related to production of whole grain crops. It appears that many of the previous publications that related to food production were excluded as they did not contain specific reference of the term “whole grains”. This may have resulted in an under-representation of studies in this important area, even though the original studies described the raw materials (e.g., unprocessed rice grains) from which whole grains and wholegrain foods are produced.

A number of observational studies had collected valuable data on wholegrain intake but whose target population (e.g., patient groups or other sub-groups) may not be entirely representative of the country’s population. Similar to the available national intake data [31,32,73], these findings tend to support that consumption of wholegrain foods is infrequent across population groups in Southeast Asia [23,24,25,27,28,29]. As in other parts of the world, there are limited consumer-centred studies in Southeast Asia aimed at understanding people’s beliefs and attitudes towards whole grains. This remains a necessary focus to consider how best to support individuals in making realistic choices that will help them move towards or meet guidelines for daily whole grain intake. The Fuldkorn public–private partnership included consumer-centred approaches in their campaigns that helped in raising average adult whole grain intake in Denmark from 36 g/d/10 MJ to 63 g/d/10 MJ [77].

It was noted that two longer-term randomized-controlled trials have been carried out in Southeast Asia which assessed the impact of switching from refined grains to whole grain alternatives (white rice versus oats and white rice versus pre-germinated brown rice respectively) [50,51]. The results have suggested benefits to health in both cases but should be interpreted with some caution in terms of their wider applicability, despite wholegrain alternatives tending to contain higher amounts of essential and non-essential nutrients than refined products. While around half of the existing published randomized, controlled trials based on increasing oat intake have suggested significant improvements in markers of cardiovascular health [78], challenges still remain when a wholegrain food is a replacement for refined alternatives made with different grain species (e.g., wholegrain oat-based foods are poor substitutes for steamed white rice in meals or dietary plans). Replacement of white rice with brown rice alternatives appears to be a means to increase wholegrain food intake through “like-for-like” substitution. However, pre-germinated rice is differentially processed, and the findings of this study should not necessarily be extrapolated to assume that all brown rice alternatives are better than refined rice, particularly as a previous meta-analyses of prospective data suggested no benefits to morbidity and mortality of increasing brown rice consumption [6]. The authors believe that a focus on wholegrain foods available in the region and that are easy to substitute for the refined alternatives is the most likely approach to increase wholegrain food intake at a population level.

Glycaemic index is an acute physiological response to carbohydrate-containing foods. Longer-term adherence to low glycaemic index and glycaemic load diets has been associated with improvements to cardiovascular and metabolic health outcomes [79]. Published glycaemic index data suggest that brown rice types have comparable values to white rice alternatives [80,81]. A number of the lowest glycaemic index values for rice have been attributed to white rice varieties [82], which may be a result of the digestibility of the starch being lower in some cultivars compared to others [83]. This highlights that the impact of rice on health cannot simply be inferred from whether it is whole grain or not. There is still a clear need for further randomized, controlled trials on the impact of whole grains relevant to the region on the health of Southeast Asians.

The Food and Agriculture Organization of the United Nations’ website includes overviews of food-based dietary guidelines in Thailand and Vietnam, although no recommendation for intake of whole grains is presented within these guidelines [84,85]. However, it appears that food-based dietary recommendations do not exist in the other ASEAN/UN member states—Brunei Darussalam, Cambodia, Lao People’s Democratic Republic and Myanmar [86,87]. The limited nationally-representative nutrition survey data in the region means that better defining absolute amounts of whole grain intake within populations is challenging [88] and has the potential to limit the development and applicability of recommendations to increase whole grain intake within member states. Target intake amounts (in grams per day) of whole grains are only currently recommended by public health agencies and non-government organisations in a handful of countries worldwide [89]. To date, most longitudinal data and randomized, controlled trials that suggest whole grain intake might benefit health outcomes have been collected in Europe and North America [15]. This situation appears to be improving in Southeast Asia, particularly in relation to cross-sectional and/or longitudinal data [31,32,73,74]. Such findings should help develop rational food-based recommendations for wholegrain consumption and help in the development of strategies to move population level intake towards this. These findings suggest that current intake of whole grains in across ASEAN is low.

As in other parts of the world, there are very few regulations in the ASEAN region that relate to definitions of whole grains and wholegrain foods. Previous calls have come from consortia of international experts to improve and standardize the definitions of wholegrain foods [90,91], which would allow dietary intake data from different countries to be more comparable, improve clarity of labelling to consumers and provide objective criteria for the food industry to follow. Recent recommendations to define a wholegrain food included minimum cut-off values for percentage of wholegrain ingredient content (≤30%) on a dry weight basis [91]. Clear definitions of what a wholegrain food is that include a minimal content of whole grains seems likely to improve consumer clarity, provide standards for the manufacturers of wholegrain foods and ensure that dietary guidelines aimed at eating more whole grains have a greater potential to lead to measurable public health benefits. The divergence is whole grain regulations noted in this work is perhaps a microcosm of the wider issues with trade harmonization across ASEAN member states [92]. The authors restate the need for and importance of clear and consistent definitions of wholegrain foods, as this is likely to form a better platform for public health messaging and product marketing around whole grains.

In 2015, China was the largest importer to and exporter from the ASEAN region [93]. Consideration of the current whole grains landscape in China therefore also seems important to ASEAN wholegrain ingredients suppliers, manufacturers, importers and exporters. There are no public health food-based dietary guidelines, although the Chinese Nutrition Society have provided such recommendations since 1989 [94]. The most recent update (The Food Guide Pagoda for Chinese Residents 2016) includes the detail “A cereal-based diet is an important characteristic of a balanced diet” with further comment that “whole grains [presumably wholegrain foods] and legumes should make up 50–150 g and tubers 50–100 g (of daily intake)” [95]. One previous study estimated the intake of “coarse grains” in Chinese adults in relation to the recommendations of the Chinese Nutrition Society, with less than 20% of individuals consuming 50 g of whole grains or more [96]. Millet and maize were the most frequently consumed grain sources, while other types of whole grain (e.g., adlay and buckwheat) that were absent or considerably less prevalent from the ASEAN studies were also noted to be eaten by Chinese participants [96].

To the authors’ knowledge, the current paper is the first to consider whole grain-related regulations, recommendations and research across the ASEAN region. It is hoped that the findings of this project will help to focus future efforts in Southeast Asia in public health, food provision and research related to whole grains. While the approach to evaluating the existing research base on whole grains in Southeast Asia provides limited potential to compare these findings to other parts of the world, it helps to qualitatively assess what expertise exists in the region. Collecting and checking information through in-country experts has the advantage of accessing content that would not be otherwise accessible to non-native speakers. However, there is still the potential that in-country regulations, recommendations and intake data related to whole grains exist but are not particularly visible. The authors sought input from in-country experts with a strong background in whole grains to try and reduce this risk and also tried to check the validity of responses wherever possible. In some cases, regulatory frameworks and public health recommendations develop or change rapidly, so it is possible that those cited in this work may be superceded in a short space of time. It was not possible to screen country-specific, non-English language literature in the same way which may have meant that important research findings were overlooked. Nonetheless, the authors feel that this approach provides a broad overview of the evidence base available on whole grains in Southeast Asia.

## 5. Conclusions

Available evidence suggests that whole grain intake is low in Southeast Asia. Extrapolating from observational data collected elsewhere in the world, increasing intake of wholegrain foods in the region therefore represents an important approach to managing the increasing burden of non-communicable diseases. Many countries are also lacking food-based dietary guidelines with clear reference to wholegrain foods which is an area that public health and bodies of national nutrition professionals should consider. Development of more country-specific longitudinal dietary intake databases in the region appears to be a rational first step to increase the research base on whole grains in the region. Consideration of dietary diversity, including the range of whole grains that are consumed in the region is also important in developing harmonized definitions and standards around wholegrain foods to ensure that the term wholegrain is appropriately presented to members of the public. One approach to achieve these goals is through public–private partnerships to increase the availability and consumer awareness of whole grains with the ultimate aim of increasing consumption at a population level.

## Figures and Tables

**Table 1:** Summary of literature search results on whole grains in Southeast Asia.

Country	Nutrition	Consumer Studies	Food Science and Technology	Food Production	Total
Brunei Darussalam	0	0	0	0	0
Cambodia	1	0	0	1	2
Indonesia	1	1	9	5	16
LPDR	0	0	0	0	0
Malaysia	5	2	18	4	29
Myanmar	0	0	0	0	0
The Philippines	6	1	6	1	14
Singapore	5	2	0	0	7
Thailand	13	4	60	5	82
Vietnam	1	0	4	2	7
Multiple countries	1	0	2	3	6
Total	33	10	99	21	163 *

* Note some of the 147 studies relate to multiple headings and/or multiple countries. LPDR = Lao People’s Democratic Republic.

**Table 2:** A summary of whole grain-related public health recommendations in the ASEAN member states.

	Questions				
Country	Are There Country-Specific, Food-Based Whole Grain Recommendations from Public Health Agencies (e.g., Ministry of Health)?	Are There Suggestions for Wholegrain Food Portion Size or Amounts of Whole Grains in These Food-Based Recommendations?	Has the Public Health Agency Suggested (Scientific or Other) Rationale for Increasing Whole Grain Intake?	Do the Above Recommendations Suggest One or More scientific/Other Rationale is Suggested for Increasing Whole Grain Intake?	Are the Public Health Agencies Using Specific Tools, Educational Materials or Other Approaches to Increase Whole Grain Intake?
Brunei					
Cambodia					
Indonesia	Y [52]	N	N	N	N
LPDR					
Malaysia	Y [53]	Y [53]	Y [53]	Y [53]	N
Myanmar					
The Philippines	Y [54]	Y [54]	Y [54]	N	Y [53]
Singapore	Y [55]	Y [56]	Y [56]	Y [57]	Y [56,58]
Thailand	N	N	N	N	N
Vietnam	N	N	N	N	N

LPDR = Lao People’s Democratic Republic.

**Table 3:** Whole grain-related recommendations from professional bodies/non-governmental organisations in the ASEAN member states. LPDR = Lao People’s Democratic Republic.

	Questions			
Country	Are There Country-Specific, Food-Based Whole Grain Recommendations From Professional Groups or Non-Governmental Organisations (e.g., Nutrition Society/Whole Grains Council etc.)?	Are There Suggestions for Wholegrain Food Portion Size or Amounts of Whole Grains In The Above Mentioned Guideline?	Do The Above Recommendations Suggest (Scientific or Other) Rationale for Increasing Whole Grain Intake?	Are the Above Professional Bodies or Non-Governmental Organisations Using Specific Tools, Educational Materials or Other Approaches to Increase Whole Grain Intake?
Brunei				
Cambodia				
Indonesia	N	N	N	N
LPDR				
Malaysia	Y [53,59]	Y [53,60]	Y [53,59]	Y [60,61,62,63]
Myanmar				
The Philippines	N	N	N	N
Singapore	Y [64]	N	Y [64]	N
Thailand	N	N	N	N
Vietnam	N	N	N	N

**Table 4:** An overview of whole-grain-related regulations and health claims specific to each ASEAN country.

	Questions				
Country	Are There Existing Regulations Related to the Definition of Whole Grains and Wholegrain Foods?	Do the Regulations Include Requirements for Minimum Amounts of Whole Grains in Various Foods?	Are There Labelling Requirements or Options for Whole Grains and Wholegrain Foods?	Do the Regulations Allow Content Claims Related to Whole Grains?	Do the Regulations Allow Any Health Claims Related to Whole Grains?
Brunei					
Cambodia					
Indonesia	Y [68]	Y [68]	Y [69]	Y [69]	N [70]
LPDR					
Malaysia	Y ^a^	Y	Y ^a^	N	N
Myanmar					
The Philippines	N	N	N	N	N
Singapore	Y [65]	N	Y [71]	Y ^b^ [66]	Y ^b^ [66]
Thailand	N	N	N	N	Y ^c^
Vietnam	N	N	N	Y^c^	Y ^c^

^a^ Draft regulations are currently receiving feedback from public consultation. These regulations require whole grain content to be displayed on pack if a claim is made. ^b^ “Nutrient specific diet-related health claims” can be made for products that qualify for labelling in the categories described in the claim (including food that are labelled “wholegrain”) but also meet low-fat/high fibre content claims. ^c^ it appears possible to make claims but this is not guided or supported by existing regulations. LPDR = Lao People’s Democratic Republic.

**Table 5:** Availability of intake data for whole grains across the ASEAN region.

Questions							
Country	Are There Estimates of Whole Grain Intake Data Available For Adults From The Last 10 Years?	Are There (Recent) Estimates of Whole Grain Intake Data Available for Children for The Last Ten Years?	Are There Data Available for Changes in Whole Grain Intake over Time for Adults?	Are There Data Available for Changes in Whole Grain Intake over Time for Children?	Are There Data on the Percentage of Adults that Meet the Public Health Recommendations (Either in Amounts or Servings) for This Country?	Are There Data on the Percentage of Children That Meet the Public Health Recommendations for This Country?	Do Data Exist (From Market or Nutritional Surveys) on the Types of Wholegrain Foods That Are Consumed and the Proportion That They Are Consumed in?
Brunei							
Cambodia							
Indonesia	N	N	N	N	N	N	N
LDPR							
Malaysia	N	Y [32]	N	N	N^a^	N	N
Myanmar							
The Philippines	Y [73]	Y [73]	N	N			Y [73]
Singapore	Y [74]	Y [31]	Y [74]	N	Y [74]	N	Y ^b^ [31]
Thailand	N	N	N	N	N	N	N
Vietnam	N	N	N	N	N	N	N

^a^ Previous studies in children ([31] for Malaysia and [30] for Singapore) have drawn comparisons with the 48 g of whole grains per day guidelines for whole grain intake recommended by the USDA [75]. ^b^ Only in children aged 6–12 years. LPDR = Lao People’s Democratic Republic.

## Supplementary Materials

The following are available online at http://www.mdpi.com/2072-6643/10/6/752/s1, Table S1: Search Criteria.

## References

[1] Food and Agriculture Organization of the United Nations Food Balance Sheets. http://www.fao.org/faostat/en/#data/FBS.

[2] ASEAN Socio-Cultural Community Department and UNICEF EAPRO (East Asia and the Pacific Regional Office) (2016). Regional Report on Nutrition in ASEAN Volume 1.

[3] ASEAN Socio-Cultural Community Department and UNICEF EAPRO (East Asia and the Pacific Regional Office) (2016). Regional Report on Nutrition in ASEAN Volume 2.

[4] International Food Policy Research Institute (2016). Global Nutrition Report 2016: From Promise to Impact: Ending Malnutrition by 2030.

[5] International Diabetes Federation. Diabetes Atlas.

[6] Aune D., Keum N., Giovannucci E., Fadnes L.T., Boffetta P., Greenwood D.C., Tonstad S., Vatten L.J., Riboli E., Norat T. (2016). Whole grain consumption and risk of cardiovascular disease, cancer, and all cause and cause specific mortality: Systematic review and dose-response meta-analysis of prospective studies. BMJ.

[7] Aune D., Norat T., Romundstad P., Vatten L.J. (2013). Whole grain and refined grain consumption and the risk of type 2 diabetes: A systematic review and dose-response meta-analysis of cohort studies. Eur. J. Epidemiol..

[8] Aune D., Chan D.S., Lau R., Vieira R., Greenwood D.C., Kampman E., Norat T. (2011). Dietary fibre, whole grains, and risk of colorectal cancer: Systematic review and dose-response meta-analysis of prospective studies. BMJ.

[9] Thielecke F., Jonnalagadda S.S. (2014). Can whole grain help in weight management?. J. Clin. Gastroenterol..

[10] O’Neil C.E., Zanovec M., Cho S.S., Nicklas T.A. (2010). Whole grain and fiber consumption are associated with lower body weight measures in US adults: National Health and Nutrition Examination Survey 1999–2004. Nutr. Res..

[11] Albertson A.M., Reicks M., Joshi N., Gugger C.K. (2016). Whole grain consumption trends and associations with body weight measures in the United States: Results from the cross sectional National Health and Nutrition Examination Survey 2001–2012. Nutr. J..

[12] Devlin N.F.C., McNulty B.A., Gibney M.J., Thielecke F., Smith H., Nugent A.P. (2013). Whole grain intakes in the diets of Irish children and teenagers. Br. J. Nutr..

[13] Galea L.M., Beck E.J., Probst Y.C., Cashman C.J. (2017). Whole grain intake of Australians estimated from a cross-sectional analysis of dietary intake data from the 2011–13 Australian Health Survey. Public Health Nutr..

[14] Mann K.D., Pearce M.S., McKevith B., Thielecke F., Seal C.J. (2015). Low whole grain intake in the UK: Results from the National Diet and Nutrition Survey rolling programme 2008–11. Br. J. Nutr..

[15] Seal C.J., Brownlee I.A. (2015). Whole-grain foods and chronic disease: Evidence from epidemiological and intervention studies. Proc. Nutr. Soc..

[16] Global Health Data Exchange Global Burden of Disease Results Tool. http://ghdx.healthdata.org/gbd-results-tool?params=gbd-api-2016-permalink/0684f896deb2d178ec1a09e4b125d3ea.

[17] Association of Southeast Asian Nations ASEAN Free Trade Area. http://asean.org/asean-economic-community/asean-free-trade-area-afta-council/.

[18] Haitao H. (2017). The role of trust in China-ASEAN relations—Towards a multi-level trust building for China and ASEAN. Int. J. China Stud..

[19] Phattarakul N., Rerkasem B., Li L.J., Wu L.H., Zou C.Q., Ram H., Sohu V.S., Kang B.S., Surek H., Kalayci M. (2012). Biofortification of rice grain with zinc through zinc fertilization in different countries. Plant Soil.

[20] Goodman M.T., Wilkens L.R., Hankin J.H., Lyu L.C., Wu A.H., Kolonel L.N. (1997). Association of soyand fiber consumption with the risk of endometrial cancer. Am. J. Epidemiol..

[21] Peterman J.N., Silka L., Bermudez O.I., Wilde P.E., Rogers B.L. (2011). Acculturation, education, nutrition education, and household composition are related to dietary practices among Cambodian refugee women in Lowell, MA. J. Am. Diet. Assoc..

[22] McCrory M.A., Jaret C.L., Kim J.H., Reitzes D.C. (2017). Dietary patterns among vietnamese and hispanic immigrant elementary school children participating in an after school program. Nutrients.

[23] Tan S.L., Juliana S., Sakinah H. (2011). Dietary compliance and its association with glycemic control among poorly controlled type 2 diabetic outpatients in Hospital Universiti Sains Malaysia. Malays. J. Nutr..

[24] Randi G., Edefonti V., Ferraroni M., La Vecchia C., Decarli A. (2010). Dietary patterns and the risk of colorectal cancer and adenomas. Nutr. Rev..

[25] Yaw Y.H., Shariff Z.M., Kandiah M., Weay Y.H., Saibul N., Sariman S., Hashim Z. (2014). Diet and physical activity in relation to weight change among breast cancer patients. Asian Pac. J. Cancer Prev..

[26] Sangthong R., Wichaidit W., McNeil E., Chongsuvivatwong V., Chariyalertsak S., Kessomboon P., Taneepanichskul S., Putwatana P., Aekplakorn W. (2012). Health behaviors among short- and long- term ex-smokers: Results from the Thai National Health Examination Survey IV, 2009. Prev. Med..

[27] Rebello S.A., Koh H., Chen C., Naidoo N., Odegaard A.O., Koh W.P., Butler L.M., Yuan J.M., Van Dam R.M. (2014). Amount, type, and sources of carbohydrates in relation to ischemic heart disease mortality in a Chinese population: A prospective cohort study. Am. J. Clin. Nutr..

[28] Ross A.B., Colega M.T., Lim A.L., Silva-Zolezzi I., Macé K., Saw S.M., Kwek K., Gluckman P., Godfrey K.M., Chong Y.S. (2015). Whole grain intake, determined by dietary records and plasma alkylresorcinol concentrations, is low among pregnant women in Singapore. Asia Pac. J. Clin. Nutr..

[29] Whitton C., Ma Y., Bastian A.C., Fen Chan M., Chew L. (2014). Fast-food consumers in singapore: Demographic profile, diet quality and weight status. Public Health Nutr..

[30] Aekplakorn W., Satheannoppakao W., Putwatana P., Taneepanichskul S., Kessomboon P., Chongsuvivatwong V., Chariyalertsak S. (2015). Dietary Pattern and Metabolic Syndrome in Thai Adults. J. Nutr. Metab..

[31] Neo J.E., Binte Mohamed Salleh S., Toh Y.X., How K.Y.L., Tee M., Mann K., Hopkins S., Thielecke F., Seal C.J., Brownlee I.A. (2016). Whole-grain food consumption in Singaporean children aged 6–12 years. J. Nutr. Sci..

[32] Norimah A.K., Koo H.C., Hamid Jan J.M., Mohd Nasir M.T., Tan S.Y., Appukutty M., Nurliyana A.R., Thielecke F., Hopkins S., Ong M.K. (2015). Whole grain intakes in the diets of Malaysian children and adolescents-findings from the MyBreakfast study. PLoS ONE.

[33] Bulatao R.M., Romero M.V. (2014). Effects of germination on the proximate composition, antioxidant property and eating quality of brown rice (*Oryza sativa* L.). Philipp. Agric. Sci..

[34] Charoenthaikij P., Jangchud K., Jangchud A., Prinyawiwatkul W., No H.K., King J.M. (2010). Physicochemical properties and consumer acceptance of wheat-germinated brown rice bread during storage time. J. Food Sci..

[35] Charoenthaikij P., Jangchud K., Jangchud A., Prinyawiwatkul W., Tungtrakul P. (2010). Germination conditions affect selected quality of composite wheat-germinated brown rice flour and bread formulations. J. Food Sci..

[36] Alice C.L.V., Wan Rosli W.I. (2015). Effects of brown rice powder addition on nutritional composition and acceptability of two selected Malaysian traditional rice-based local *Kuih*. Int. Food Res. J..

[37] Lestari L.A., Huriyati E., Marsono Y. (2017). The development of low glycemic index cookie bars from foxtail millet (*Setaria italica*), arrowroot (*Maranta arundinacea*) flour, and kidney beans (*Phaseolus vulgaris*). J. Food Sci. Technol..

[38] Koo H.C., Poh B.K., Ruzita A.T. (2016). Development, validity and reliability of a questionnaire on knowledge, attitude and practice (KAP) towards whole grain among primary school children in Kuala Lumpur, Malaysia. Int. Food Res. J..

[39] Neo J.E., Brownlee I.A. (2017). Wholegrain food acceptance in young Singaporean adults. Nutrients.

[40] Singhato A., Banjong O., Charoonruk G. (2017). Effectiveness and acceptance of the developed educational media on the application of a Thai ethnic snack, Thong Pub, with calcium fortification. J. Ethn. Foods.

[41] Son J.S., Do V.B., Kim K.O., Cho M.S., Suwonsichon T., Valentin D. (2013). Consumers’ attitude towards rice cooking processes in Korea, Japan, Thailand and France. Food Qual. Preference.

[42] Shiu L.K.C., Loke W.M., Vijaya K., Sandhu N.K. (2012). Nurturing healthy dietary habits among children and youth in Singapore. Asia Pac. J. Clin. Nutr..

[43] Mohd Yusof B.N., Abd Talib R., Karim N.A., Kamarudin N.A., Arshad F. (2009). Glycaemic index of four commercially available breads in Malaysia. Int. J. Food Sci. Nutr..

[44] Panlasigui L.N., Thompson L.U. (2006). Blood glucose lowering effects of brown rice in normal and diabetic subjects. Int. J. Food Sci. Nutr..

[45] Se C.H., Chuah K.A., Mishra A., Wickneswari R., Karupaiah T. (2016). Evaluating crossbred red rice variants for postprandial glucometabolic responses: A comparison with commercial varieties. Nutrients.

[46] Trinidad T.P., Mallillin A.C., Sagum R.S., Felix A.D.R., Tuaño A.P.P., Juliano B.O. (2014). Relative effect of apparent amylose content on the glycemic index of milled and brown rice. Philipp. Agric. Sci..

[47] Golloso-Gubat M.J., Magtibay E.V.J., Nacis J.S., Udarbe M.A., Santos N.L.C., Timoteo V.J.A. (2016). Postprandial satiety responses and ghrelin levels with consumption of white rice and brown rice in selected Filipino adults. Philipp. J. Sci..

[48] Trinidad T.P., Kurilich A.C., Mallillin A.C., Walcyzk T., Sagum R.S., Singh N.N., Harjani Y., De Leon M.P., Capanzana M.V., Fletcher J. (2014). Iron absorption from NaFeEDTA-fortified oat beverages with or without added vitamin C. Int. J. Food Sci. Nutr..

[49] Trinidad T.P., Mallillin A.C., Sagum R.S., Briones D.P., Encabo R.R., Juliano B.O. (2009). Iron absorption from brown rice/brown rice-based meal and milled rice/milled rice-based meal. Int. J. Food Sci. Nutr..

[50] Thongoun P., Pavadhgul P., Bumrungpert A., Satitvipawee P., Harjani Y., Kurilich A. (2013). Effect of oat consumption on lipid profiles in hypercholesterolemic adults. J. Med. Assoc. Thail..

[51] Bui T.N., Le Hop T., Nguyen D.H., Tran Q.B., Nguyen T.L., Le D.T., Nguyen D.V.A., Vu A.L., Aoto H., Okuhara Y. (2014). Pre-germinated brown rice reduced both blood glucose concentration and body weight in vietnamese women with impaired glucose tolerance. J. Nutr. Sci. Vitaminol..

[52] Kementerian Kesehatan (Indonesian Ministry of Health) (2014). Pedoman Gizi Seimbang (Nutritional Guidelines Handbook).

[53] Ministry of Health Malaysia (2010). Malaysian Dietary Guidelines Key Message 4—Eat Adequate Amount of Rice, Other Cereal Products (Preferably Whole Grain) and Tubers.

[54] Republic of the Philippines Food and Nutrition Research Institute (2016). Pinggang Pinoy (Filipino Plate).

[55] Health Promotion Board My Healthy Plate. https://www.healthhub.sg/programmes/55/my-healthy-plate.

[56] Health Promotion Board Whole Grains—The Wise Choice!. https://www.healthhub.sg/live-healthy/183/whole_grains_wise_choice.

[57] Health Promotion Board Food-Based Dietary Guidelines for Adults. https://www.healthhub.sg/live-healthy/15/dietary_guidelines_adults.

[58] Health Promotion Board (2018). HealthHub Track.

[59] Nutrition Society of Malaysia Dietary Guidelines. http://nsm.nutritionmonthmalaysia.org.my/dietary-guidelines/.

[60] Nutrition Society of Malaysia (2012). Wonders of Whole Grains.

[61] Nutrition Society of Malaysia Eat Smart, Get Fit & Feel Great. An infographic Kit—Nutrition Month Malaysia. http://nutritionmonthmalaysia.org.my/wp-content/uploads/2017/09/NMM%202016%20Info%20Graphic%20Book%20(Final).pdf.

[62] Nutrition Society of Malaysia Eat Smart, Move More. Recipe for Healthy Families. An Infographic Kit Volume 2—Nutrition Month Malaysia..

[63] Nutrition Society of Malaysia Your Only Choice: Eat Healthy, Be Active. An Infographic Kit—Nutrition Month Malaysia. http://nutritionmonthmalaysia.org.my/wp-content/uploads/2018/04/NMM%202018%20Info%20Graphic%20Book%20(FINAL%20PRINT).pdf.

[64] Singapore Nutrition and Dietetic Association, Health Promotion Board (2016). Advocating Whole Grains Consumption as a Key Approach in Diabetes Prevention.

[65] Agri-Food and Veterinary Agency of Singapore (2017). Sale of Food Act (Chapter 283, Section 56(1)) Food Regulations.

[66] Agri-Food and Veterinary Agency of Singapore (2017). A Guide to Food Labelling and Advertisements.

[67] Ministry of Health Malaysia (2018). Online Public Engagement No 1/2018 Proposed Draft Amendment for Food Regulation 1985.

[68] Badan Pengawas Obat Dan Makanan Republik Indonesia (Republic of Indonesia’s National Agency of Drug and Food Control) (2016). Peraturan Kepala Badan Pengawas Obat Dan Makanan Nomor 21 (Regulation Number 21 of the Chief of the National Agency of Drug and Food Control).

[69] Badan Pengawas Obat Dan Makanan Republik Indonesia (Republic of Indonesia’s National Agency of Drug and Food Control) (2016). Peraturan Kepala Badan Pengawas Obat Dan Makanan Nomor 12 (Regulation Number 12 of the Chief of the National Agency of Drug and Food Control Number 12).

[70] Badan Pengawas Obat Dan Makanan Republik Indonesia (Republic of Indonesia’s National Agency of Drug and Food Control) (2016). Peraturan Kepala Badan Pengawas Obat Dan Makanan Nomor 13 (Regulation Number 13 of the Chief of the National Agency of Drug and Food Control Number 12).

[71] Health Promotion Board (2018). Healthier Choice Symbol Nutrient Guidelines.

[72] National Bureau of Agricultural Commodity and Food Standards of Thailand Database of Food Consumption of Thai People. http://consumption.acfs.go.th/index.php.

[73] Republic of the Philippines Food and Nutrition Research Institute (2013). 8th National Nutrition Survey.

[74] Health Promotion Board (2010). National Nutrition Survey.

[75] US Department of Health (Human Services) (2017). Dietary Guidelines for Americans 2015–2020.

[76] Wailes E.J., Chavez E.C. (2012). ASEAN and the Global Rice Situation and Outlook.

[77] Greve C., Neess R.I. (2014). The Evolution of the Whole Grain Partnership in Denmark.

[78] Thies F., Masson L.F., Boffetta P., Kris-Etherton P. (2014). Oats and CVD risk markers: A systematic literature review. Br. J. Nutr..

[79] Louie J.C.Y., Jones M., Barclay A.W., Brand-Miller J.C. (2017). Dietary glycaemic index and glycaemic load among Australian adults-results from the 2011–2012 Australian Health Survey. Sci. Rep..

[80] Atkinson F.S., Foster-Powell K., Brand-Miller J.C. (2008). International tables of glycemic index and glycemic load values: 2008. Diabetes Care.

[81] Kaur B., Ranawana V., Henry J. (2016). The Glycemic Index of Rice and Rice Products: A Review, and Table of GI Values. Crit. Rev. Food Sci. Nutr..

[82] Ranawana D.V., Henry C.J.K., Lightowler H.J., Wang D. (2009). Glycaemic index of some commercially available rice and rice products in Great Britain. Int. J. Food Sci. Nutr..

[83] Hu P., Zhao H., Duan Z., Linlin Z., Wu D. (2004). Starch digestibility and the estimated glycemic score of different types of rice differing in amylose contents. J. Cereal Sci..

[84] Food and Agricultural Organization of the United Nations Food-Based Dietary Guidelines—Thailand. http://www.fao.org/nutrition/education/food-based-dietary-guidelines/regions/countries/thailand/en/.

[85] Food and Agricultural Organization of the United Nations Food-Based Dietary Guidelines—Vietnam. http://www.fao.org/nutrition/education/food-based-dietary-guidelines/regions/countries/vietnam/en/.

[86] Food and Agricultural Organization of the United Nations Food-Based Dietary Guidelines—Asia and the Pacific. http://www.fao.org/nutrition/education/food-dietary-guidelines/regions/asia-pacific/en/.

[87] Tee E., Hardinsyah R., Fiorentino R., Ismail M., Suthutvoravut U., Hop L. (2016). Food-based dietary guidelines of Southeast Asian countries: Part 1-A compilation and analysis of key messages. Malays. J. Nutr..

[88] Ross A.B., Kristensen M., Seal C.J., Jacques P., McKeown N.M. (2015). Recommendations for reporting whole-grain intake in observational and intervention studies. Am. J. Clin. Nutr..

[89] Seal C.J., Nugent A.P., Tee E.S., Thielecke F. (2016). Whole-grain dietary recommendations: The need for a unified global approach. Br. J. Nutr..

[90] Korczak R., Marquart L., Slavin J.L., Ringling K., Chu Y., O’Shea M., Harriman C., Toups K., De Vries J., Jacques P. (2016). Thinking critically about whole-grain definitions: Summary report of an interdisciplinary roundtable discussion at the 2015 Whole Grains Summit1. Am. J. Clin. Nutr..

[91] Ross A.B., van der Kamp J.W., King R., Lê K.A., Mejborn H., Seal C.J., Thielecke F. (2017). Perspective: A definition for whole-grain food products—Recommendations from the Healthgrain Forum. Adv. Nutr..

[92] Devadason E.S., Chandran V.G.R., Kalirajan K. (2018). Harmonization of food trade standards and regulations in ASEAN: The case of Malaysia’s food imports. Agric. Econ..

[93] Association of Southeast Asian Nations (2016). External Trade Statistics: Top Ten ASEAN Trade Partner Countries/Regions 2015.

[94] Food and Agricultural Organization of the United Nations Food-Based Dietary Guidelines—China. http://www.fao.org/nutrition/education/food-dietary-guidelines/regions/asia-pacific/en/.

[95] Chinese Nutrition Society (2016). Chinese Dietary Guidelines 2016.

[96] He Y., Zhao L., Yu D., Hu J., Yang Y., Yang X. (2016). The status of coarse food intakes among Chinese adults. Acta Nutr. Sin..

