# Supplementary material for: An Overview of Whole Grain Regulations, Recommendations and Research across Southeast Asia
Source: Nutrients. 2018 Jun 11;10(6):752. doi: 10.3390/nu10060752 (PMC6024883; doi:10.3390/nu10060752)
Supplement: Supplementary file 1 [file nutrients-10-00752-s001.zip › nutrients-310352-supplementary.pdf]

Supplementary Materials 1

Table S1. Search criteria.

**Whole grain-related search terms**		**Country-specific terms**
whole grain OR whole grains OR wholegrain OR wholemeal		"Thai" OR "Thailand"
"barley"		"Myanmar" OR "Burm*"
"maize" OR "corn"		"Brunei" OR "Bruneian"
"millet"		"Malay*"
"oats" OR "oat"	AND	"Singapor*"
"brown rice" OR "black rice" OR "red rice" OR "wild rice"		"Cambod*"
"rye"		"Viet Nam" or "Viet*"
"sorghum"		"Indones*"
"whole wheat" OR "wholewheat" OR "*wheat"		"Lao" OR "Laos" OR "Laotian"
		"Filipin*" or "Philippines"

List of published, whole grain- and ASEAN-related studies

Brunei

No articles

Cambodia

1. Meas, P.; Paterson, A.H.J.; Cleland, D.J.; Bronlund, J.E.; Mawson, A.J.; Hardacre, A.; Rickman, J.F. Measurement of bed grain and air conditions during solar drying of rice. *International Journal of Food Engineering* **2011**, *7*.
2. Peterman, J.N.; Silka, L.; Bermudez, O.I.; Wilde, P.E.; Rogers, B.L. Acculturation, education, nutrition education, and household composition are related to dietary practices among Cambodian refugee women in Lowell, MA. *Journal of the American Dietetic Association* **2011**, *111*, 1369-1374.

Indonesia

3. Anggraini, T.; Novelina; Limber, U.; Amelia, R. Antioxidant activities of some red, black and white rice cultivar from west sumatra, Indonesia. *Pakistan Journal of Nutrition* **2015**, *14*, 112-117.
4. Fidrianny, I.; Puspitaningrum, D.A.; Ruslan, K. Antioxidant capacities of various grains extracts of three kinds of rice grown in Central Java-Indonesia. *International Journal of Pharmacognosy and Phytochemical Research* **2016**, *8*, 997-1002.
5. Jati, I.R.A.P.; Nohr, D.; Biesalski, H.K. Nutrients and antioxidant properties of Indonesian underutilized colored rice. *Nutrition and Food Science* **2014**, *44*, 193-203.
6. Lestari, L.A.; Huriyati, E.; Marsono, Y. The development of low glycemic index cookie bars from foxtail millet (Setaria italica), arrowroot (Maranta arundinacea) flour, and kidney beans (Phaseolus vulgaris). *Journal of Food Science and Technology* **2017**, *54*, 1406-1413.
7. Moko, E.M.; Purnomo, H.; Kusnadi, J.; Ijong, F.G. Phytochemical content and antioxidant properties of colored and non colored varieties of rice bran from Minahasa, North Sulawesi, Indonesia. *International Food Research Journal* **2014**, *21*, 1017-1023.

8. Murdifin, M.; Pakki, E.; Rahim, A.; Syaiful, S.A.; Ismail; Evary, Y.M.; Akbar Bahar, M. Physicochemical properties of indonesian pigmented rice (Oryza sativa linn.) varieties from south sulawesi. *Asian Journal of Plant Sciences* **2015**, *14*, 59-65.
9. Sabir, A.; Rafi, M.; Darusman, L.K. Discrimination of red and white rice bran from Indonesia using HPLC fingerprint analysis combined with chemometrics. *Food Chemistry* **2017**, *221*, 1717-1722.
10. Setyaningsih, W.; Hidayah, N.; Saputro, I.E.; Palma, M.; García Barroso, C. Profile of phenolic compounds in Indonesian rice (Oryza sativa) varieties throughout post-harvest practices. *Journal of Food Composition and Analysis* **2016**, *54*, 55-62.
11. Sukrasno, S.; Tuty, S.; Fidrianny, I. Antioxidant evaluation and phytochemical content of various rice bran extracts of three varieties rice from Semarang, Central Java, Indonesia. *Asian Journal of Pharmaceutical and Clinical Research* **2017**, *10*, 377-382.
12. Mau, Y.S.; Markus, J.E.R.; Shirly; Oematan, S.; Ndiwa, A.S.S.; Handoko, D.D.; Nasution, A.; Makbul, K. Genetic diversity of red and black upland rice accessions from east nusa tenggara, Indonesia as revealed by agro-morphological characters. *Biodiversitas* **2017**, *18*, 197-211.
13. Muliarta Aryana, I.G.P.; Wangiyana, W. Yield performance and adaptation of promising amphibious red rice lines on six growing environments in Lombok, Indonesia. *Agrivita* **2016**, *38*, 40-46.
14. Mustikarini, E.D.; Ardiarini, N.R.; Basuki, N.; Kuswanto, K. Selection strategy of drought tolerance on red rice mutant lines. *Agrivita* **2017**, *39*, 91-99.
15. Ruminta; Nurmala, T.; Wicaksono, F.Y. Growth and yield of job's tears (Coix lacryma-jobi L.) response to different types of oldeman climate classification and row spacing in west java Indonesia. *Journal of Agronomy* **2017**, *16*, 76-82.
16. Suliartini, N.W.S.; Kuswanto, K.; Basuki, N.; Soegianto, A. Superior lines candidates evaluation of two local red rice Southeast Sulawesi cultivars (Indonesia) derived from gamma rays irradiation techniques. *International Journal of Plant Biology* **2016**, *7*, 64-67.

Lao Democratic People's Republic

No articles

Malaysia

17. Abubakar, B.; Yakasai, H.M.; Zawawi, N.; Ismail, M. Compositional analyses of white, brown and germinated forms of popular Malaysian rice to offer insight into the growing diet-related diseases. *Journal of Food and Drug Analysis* **2017**.
18. Alice, C.L.V.; Wan Rosli, W.I. Effects of brown rice powder addition on nutritional composition and acceptability of two selected Malaysian traditional rice-based local kuih. *International Food Research Journal* **2015**, *22*, 1124-1131.
19. Chew, S.C.; Loh, S.P.; Khor, G.L. Determination of folate content in commonly consumed Malaysian foods. *International Food Research Journal* **2012**, *19*, 189-197.
20. Fasahat, P.; Muhammad, K.; Abdullah, A.; Ratnam, W. Proximate nutritional composition and antioxidant properties of Oryza rufipogon, a wild rice collected from Malaysia compared to cultivated rice, MR219. *Australian Journal of Crop Science* **2012**, *6*, 1502-1507.
21. Ibrahim, S.; Abdul Rahim, H. In *The assessment of amylose content using near infrared spectroscopy analysis of rice grain samples*, 2013 IEEE Conference on Open Systems, ICOS 2013, 2013; pp 32-37.
22. Chung, I.M.; Kim, J.K.; Prabakaran, M.; Yang, J.H.; Kim, S.H. Authenticity of rice (Oryza sativa L.) geographical origin based on analysis of C, N, O and S stable isotope ratios: A preliminary case report in Korea, China and Philippine. *Journal of the Science of Food and Agriculture* **2016**, *96*, 2433-2439.
23. Lim, S.M.; Goh, Y.M.; Loh, S.P. Nutritional Compositions and antioxidant activities of non-polar and polar extracts of germinated brown rice. Pertanika Journal of Tropical Agricultural Science **2016**, *39*, 219-233.

24. Mohd-Redzwan, S.; Jamaluddin, R.; Abd-Mutalib, M.S.; Ahmad, Z. A mini review on aflatoxin exposure in Malaysia: Past, present, and future. *Frontiers in Microbiology* **2013**, *4*.
25. Musa, A.S.N.; Umar, I.M.; Ismail, M. Physicochemical properties of germinated brown rice (Oryza sativa L.) starch. *African Journal of Biotechnology* **2011**, *10*, 6281-6291.
26. Norhayati, M.K.; Mohd Fairulnizal, M.N.; Zaiton, A.; Wan Syuriahti, W.Z.; Rusidah, S.; Aswir, A.R.; Ang, J.L.; Mohd Naeem, M.N.; Suraiami, M.; Mohd Azerulazree, J.*, et al.* Nutritional composition of selected commercial biscuits in Malaysia. *Sains Malaysiana* **2015**, *44*, 581-591.
27. Rahim, H.A.; Ibrahim, S. Using near-infrared spectroscopy to investigate the amylose content in rice. In *Applied Mechanics and Materials*, 2013; Vol. 239-240, pp 163-166.
28. Reddy, K.R.N.; Salleh, B. A preliminary study on the occurrence of Aspergillus spp. and aflatoxin B<inf>1</inf> in imported wheat and barley in Penang, Malaysia. *Mycotoxin Research* **2010**, *26*, 267-271.
29. Roohinejad, S.; Mirhosseini, H.; Saari, N.; Mustafa, S.; Alias, I.; Meor Hussin, A.S.; Hamid, A.; Manap, M.Y. Evaluation of GABA, crude protein and amino acid composition from different varieties of Malaysian's brown rice. *Australian Journal of Crop Science* **2009**, *3*, 184-190.
30. Roohinejad, S.; Omidizadeh, A.; Mirhosseini, H.; Saari, N.; Mustafa, S.; Meor Hussin, A.S.; Hamid, A.; Abd Manap, M.Y. Effect of pre-germination time on amino acid profile and gamma amino butyric acid (GABA) contents in different varieties of Malaysian brown rice. *International Journal of Food Properties* **2011**, *14*, 1386-1399.
31. Samsudin, N.I.P.; Abdullah, N. A preliminary survey on the occurrence of mycotoxigenic fungi and mycotoxins contaminating red rice at consumer level in Selangor, Malaysia. *Mycotoxin Research* **2013**, *29*, 89-96.
32. Sawei, J.; Sani, N.A. In *Prevalence, isolation and characterization of Bacillus cereus strains from rice of local cultivators of Sabah, Sarawak, and Peninsular Malaysia*, AIP Conference Proceedings, 2016.
33. Shammugasamy, B.; Ramakrishnan, Y.; Ghazali, H.M.; Muhammad, K. Tocopherol and tocotrienol contents of different varieties of rice in Malaysia. *Journal of the Science of Food and Agriculture* **2014**.
34. Chambers, E.S.; Viardot, A.; Psichas, A.; Morrison, D.J.; Murphy, K.G.; Zac-Varghese, S.E.K.; MacDougall, K.; Preston, T.; Tedford, C.; Finlayson, G.S.*, et al.* Effects of targeted delivery of propionate to the human colon on appetite regulation, body weight maintenance and adiposity in overweight adults. *Gut* **2015**, *64*, 1744-1754.
35. Gordon, M.; Naidoo, K.; Akobeng, A.K.; Thomas, A.G. Cochrane Review: Osmotic and stimulant laxatives for the management of childhood constipation. *Evidence-Based Child Health* **2013**, *8*, 57-109.
36. Ambak, K.; Tadano, T.; Ambak, K. Effect of micronutrient application on the growth and occurrence of sterility in barley and rice in a Malaysian deep peat soil. *Soil Science and Plant Nutrition* **1991**, *37*, 715-724.
37. Cui, Y.; Song, B.K.; Li, L.F.; Li, Y.L.; Huang, Z.; Caicedo, A.L.; Jia, Y.; Olsen, K.M. Little white lies: Pericarp color provides insights into the origins and evolution of Southeast Asian weedy rice. *G3: Genes, Genomes, Genetics* **2016**, *6*, 4105-4114.
38. Koo, H.C.; Poh, B.K.; Ruzita, A.T. Development, validity and reliability of a questionnaire on knowledge, attitude and practice (KAP) towards whole grain among primary school children in Kuala Lumpur, Malaysia. *International Food Research Journal* **2016**, *23*, 797-805.
39. Mohd Yusof, B.N.; Abd. Talib, R.; Karim, N.A.; Kamarudin, N.A.; Arshad, F. Glycaemic index of four commercially available breads in Malaysia. *International Journal of Food Sciences and Nutrition* **2009**, *60*, 487-496.
40. Norimah, A.K.; Koo, H.C.; Hamid Jan, J.M.; Mohd Nasir, M.T.; Tan, S.Y.; Appukutty, M.; Nurliyana, A.R.; Thielecke, F.; Hopkins, S.; Ong, M.K.*, et al.* Whole grain intakes in the diets of

Malaysian children and adolescents-findings from the MyBreakfast study. *PLoS ONE* **2015**, *10*.
41. Song, B.K.; Chuah, T.S.; Tam, S.M.; Olsen, K.M. Malaysian weedy rice shows its true stripes: Wild Oryza and elite rice cultivars shape agricultural weed evolution in Southeast Asia. *Molecular Ecology* **2014**, *23*, 5003-5017.
42. Alsaffar, A.A. Effect of food processing on the resistant starch content of cereals and cereal products - a review. *International Journal of Food Science and Technology* **2011**, *46*, 455-462.
43. Yaw, Y.H.; Shariff, Z.M.; Kandiah, M.; Weay, Y.H.; Saibul, N.; Sariman, S.; Hashim, Z. Diet and physical activity in relation to weight change among breast cancer patients. *Asian Pacific Journal of Cancer Prevention* **2014**, *15*, 39-44.

Multiple countries

44. Sumczynski, D.; Kotásková, E.; Družbíková, H.; Mlček, J. Determination of contents and antioxidant activity of free and bound phenolics compounds and in vitro digestibility of commercial black and red rice (Oryza sativa L.) varieties. *Food Chemistry* **2016**, *211*, 339-346.
45. Chapagai, M.K.; Wan Rosli, W.I.; Wan Manan, W.M.; Jalil, R.A.; Karrila, T.; Pinkaew, S. Effect of domestic cooking methods on physicochemical, nutritional and sensory properties of different varieties of brown rice from Southern Thailand and Malaysia. *International Food Research Journal* **2017**, *24*, 1140-1147.
46. Muhidin; Kamaruzaman, J.; Elwakib, S.; Yunus, M.; Kaimuddin; Meisanti, A.; Ray, S.G.; La Rianda, B. The development of upland red rice under shade trees. *World Applied Sciences Journal* **2013**, *24*, 23-30.
47. Phattarakul, N.; Rerkasem, B.; Li, L.J.; Wu, L.H.; Zou, C.Q.; Ram, H.; Sohu, V.S.; Kang, B.S.; Surek, H.; Kalayci, M.*, et al.* Biofortification of rice grain with zinc through zinc fertilization in different countries. *Plant and Soil* **2012**, *361*, 131-141.
48. Agbaje, R.; Hassan, C.; Norlelawati, A.; Abdul Rahman, A.; Huda-Faujan, N. Development and physico-chemical analysis of granola formulated with puffed glutinous rice and selected dried Sunnah foods. *International Food Research Journal* **2016**, *23*.

Myanmar

No articles

The Philippines

49. Bulatao, R.M.; Romero, M.V. Effects of germination on the proximate composition, antioxidant property and eating quality of brown rice (oryza sativa L.). *Philippine Agricultural Scientist* **2014**, *97*, 19-27.
50. Danbaba, N.; Anounye, J.; Gana, A.; Abo, M.; Ukwungwu, M. Grain quality characteristics of Ofada rice (Oryza sativa L.): Cooking and eating quality. *International Food Research Journal* **2011**, *18*.
51. Hunt, J.R.; Johnson, L.K.; Juliano, B.O. Bioavailability of zinc from cooked Philippine milled, undermilled, and brown rice, as assessed in rats by using growth, bone zinc, and zinc-65 retention. *Journal of Agricultural and Food Chemistry* **2002**, *50*, 5229-5235.
52. Juliano, B.O.; Perez, C.M.; Resurreccion, A.P. Apparent amylose content and gelatinization temperature types of Philippine rice accessions in the IRRI gene bank. *Philippine Agricultural Scientist* **2009**, *92*, 106-109.
53. O'Dell, B.L.; De Boland, A.R.; Koirtyohann, S.R. Distribution of phytate and nutritionally important elements among the morphological components of cereal grains. *Journal of Agricultural and Food Chemistry* **1972**, *20*, 718-723.
54. Villareal, C.P.; Juliano, B.O. Variability in contents of thiamine and riboflavin in brown rice, crude oil in brown rice and bran-polish, and silicon in hull of IR rices. *Plant Foods for Human Nutrition* **1989**, *39*, 287-297.

55. Golloso-Gubat, M.J.; Magtibay, E.V.J.; Nacis, J.S.; Udarbe, M.A.; Santos, N.L.C.; Timoteo, V.J.A. Postprandial satiety responses and ghrelin levels with consumption of white rice and brown rice in selected Filipino adults. *Philippine Journal of Science* **2016**, *145*, 405-412.
56. Goodman, M.T.; Wilkens, L.R.; Hankin, J.H.; Lyu, L.C.; Wu, A.H.; Kolonel, L.N. Association of soy and fiber consumption with the risk of endometrial cancer. *American Journal of Epidemiology* **1997**, *146*, 294-306.
57. Trinidad, T.P.; Kurilich, A.C.; Mallillin, A.C.; Walcyzk, T.; Sagum, R.S.; Singh, N.N.; Harjani, Y.; De Leon, M.P.; Capanzana, M.V.; Fletcher, J. Iron absorption from NaFeEDTA-fortified oat beverages with or without added vitamin C. *International Journal of Food Sciences and Nutrition* **2014**, *65*, 124-128.
58. Trinidad, T.P.; Mallillin, A.C.; Sagum, R.S.; Briones, D.P.; Encabo, R.R.; Juliano, B.O. Iron absorption from brown rice/brown rice-based meal and milled rice/milled rice-based meal. *International Journal of Food Sciences and Nutrition* **2009**, *60*, 688-693.
59. Trinidad, T.P.; Mallillin, A.C.; Sagum, R.S.; Felix, A.D.R.; Tuaño, A.P.P.; Juliano, B.O. Relative effect of apparent amylose content on the glycemic index of milled and brown rice. *Philippine Agricultural Scientist* **2014**, *97*, 405-408.

Singapore

60. Neo, J.E.; Binte Mohamed Salleh, S.; Toh, Y.X.; How, K.Y.L.; Tee, M.; Mann, K.; Hopkins, S.; Thielecke, F.; Seal, C.J.; Brownlee, I.A. Whole-grain food consumption in Singaporean children aged 6–12 years. *Journal of Nutritional Science* **2016**.
61. Neo, J.E.; Brownlee, I.A. Wholegrain food acceptance in young Singaporean adults. *Nutrients* **2017**, *9*.
62. Hasjim, J.; Lee, S.O.; Hendrich, S.; Setiawan, S.; Ai, Y.; Jane, J.L. Characterization of a novel resistant-starch and its effects on postprandial plasma-glucose and insulin responses. *Cereal Chemistry* **2010**, *87*, 257-262.
63. Rebello, S.A.; Koh, H.; Chen, C.; Naidoo, N.; Odegaard, A.O.; Koh, W.P.; Butler, L.M.; Yuan, J.M.; Van Dam, R.M. Amount, type, and sources of carbohydrates in relation to ischemic heart disease mortality in a Chinese population: A prospective cohort study. *American Journal of Clinical Nutrition* **2014**, *100*, 53-64.
64. Adam, C.L.; Williams, P.A.; Garden, K.E.; Thomson, L.M.; Ross, A.W. Dose-dependent effects of a soluble dietary fibre (pectin) on food intake, adiposity, gut hypertrophy and gut satiety hormone secretion in rats. *PLoS ONE* **2015**, *10*.
65. Shiu, L.K.C.; Loke, W.M.; Vijaya, K.; Sandhu, N.K. Nurturing healthy dietary habits among children and youth in Singapore. *Asia Pacific Journal of Clinical Nutrition* **2012**, *21*, 144-150.
66. Whitton, C.; Ma, Y.; Bastian, A.C.; Fen Chan, M.; Chew, L. Fast-food consumers in singapore: Demographic profile, diet quality and weight status. *Public Health Nutrition* **2014**, *17*, 1805-1813.

Thailand

67. Charoenthaikij, P.; Jangchud, K.; Jangchud, A.; Prinyawiwatkul, W.; No, H.K.; King, J.M. Physicochemical properties and consumer acceptance of wheat-germinated brown rice bread during storage time. *Journal of Food Science* **2010**, *75*, S333-S339.
68. Charoenthaikij, P.; Jangchud, K.; Jangchud, A.; Prinyawiwatkul, W.; Tungtrakul, P. Germination conditions affect selected quality of composite wheat-germinated brown rice flour and bread formulations. *Journal of Food Science* **2010**, *75*, S312-S318.
69. Inglett, G.E.; Carriere, C.J.; Maneepun, S.; Boonpunt, T. Nutritional value and functional properties of a hydrocolloidal soybean and oat blend for use in Asian foods. *Journal of the Science of Food and Agriculture* **2003**, *83*, 86-92.
70. Inglett, G.E.; Carriere, C.J.; Maneepun, S.; Tungtrakul, P. A soluble fibre gel produced from rice bran and barley flour as a fat replacer in Asian foods. *International Journal of Food Science and Technology* **2004**, *39*, 1-10.

71. Parnsakhorn, S.; Noomhorm, A. Effects of storage temperature on physical and chemical properties of brown rice, parboiled brown rice and parboiled paddy. *Thai Journal of Agricultural Science* **2012**, *45*, 221-231.
72. Sangkitikomol, W.; Tencomnao, T.; Rocejanasaroj, A. Antioxidant effects of anthocyanins-rich extract from black sticky rice on human erythrocytes and mononuclear leukocytes. *African Journal of Biotechnology* **2010**, *9*, 8222-8229.
73. Sangkitikomol, W.; Tencomnao, T.; Rocejanasaroj, A. Effects of thai black sticky rice extract on oxidative stress and lipid metabolism gene expression in hepg2 cells. *Genetics and Molecular Research* **2010**, *9*, 2086-2095.
74. Sompong, R.; Siebenhandl-Ehn, S.; Berghofer, E.; Schoenlechner, R. Extrusion cooking properties of white and coloured rice varieties with different amylose content. *Starch/Staerke* **2011**, *63*, 55-63.
75. Watchararparpaiboon, W.; Laohakunjit, N.; Kerdchoechuen, O. An improved process for high quality and nutrition of brown rice production. *Food Science and Technology International* **2010**, *16*, 147-158.
76. Attaviroj, N.; Kasemsumran, S.; Noomhorm, A. Rapid variety identification of pure rough rice by fourier-transform near-infrared spectroscopy. *Cereal Chemistry* **2011**, *88*, 490-496.
77. Imsil, A.; Rittiron, R.; Sirisomboon, P.; Areekul, V. Classification of Hom Mali rice with different degrees of milling based on physicochemical measurements by principal component analysis. *Kasetsart Journal - Natural Science* **2011**, *45*, 863-873.
78. Inglett, G.E.; Maneepun, S.; Vatanasuchart, N. Evaluation of hydrolyzed oat flour as a replacement for butter and coconut cream in bakery products Evaluación del uso de harina de avena en sustitución de la mantequilla y crema de coco en productos de pastelería. *Food Science and Technology International* **2000**, *6*, 457-462.
79. Keawpeng, I.; Meenune, M. Changes in cooking behavior of organic and inorganic phatthalung sungyod rice during ageing. *Thai Journal of Agricultural Science* **2011**, *44*, 348-353.
80. Suntaro, K.; Sangchum, K.; Tirawanichakul, S.; Tirawanichakul, Y. Artificial neural network approach for impingement drying of germinated brown rice soaking with turmeric solution. In *Applied Mechanics and Materials*, 2013; Vol. 372, pp 463-466.
81. Banchuen, J.; Thammarutwasik, P.; Ooraikul, B.; Wuttijumnong, P.; Sivongpaisal, P. Increasing the bio-active compounds contents by optimizing the germination conditions of southern Thai brown rice. *Songklanakarin Journal of Science and Technology* **2010**, *32*, 219-230.
82. Banjerdpongchai, R.; Wudtiwai, B.; Sringarm, K. Cytotoxic and apoptotic-inducing effects of purple rice extracts and chemotherapeutic drugs on human cancer cell lines. *Asian Pacific Journal of Cancer Prevention* **2013**, *14*, 6541-6548.
83. Bansal, J.; Pantazopoulos, P.; Tam, J.; Cavlovic, P.; Kwong, K.; Turcotte, A.M.; Lau, B.P.Y.; Scott, P.M. Surveys of rice sold in canada for aflatoxins, ochratoxin a and fumonisins. *Food Additives and Contaminants - Part A Chemistry, Analysis, Control, Exposure and Risk Assessment* **2011**, *28*, 767-774.
84. Butsat, S.; Siriamornpun, S. Antioxidant capacities and phenolic compounds of the husk, bran and endosperm of Thai rice. *Food Chemistry* **2010**, *119*, 606-613.
85. Chinprahast, N.; Tungsomboon, T.; Nagao, P. Antioxidant activities of Thai pigmented rice cultivars and application in sunflower oil. *International Journal of Food Science and Technology* **2016**, *51*, 46-53.
86. Chooklin, S. Ultrasound-assisted extraction of phenolic compounds from brown rice and their antioxidant activities. *Kasetsart Journal - Natural Science* **2013**, *47*, 864-873.
87. Fasahat, P.; Abdullah, A.; Muhammad, K.; Musa, K.H.; Wickneswari, R. New red rice transgressive variants with high antioxidant capacity. *International Food Research Journal* **2013**, *20*, 1497-1501.

88. Jannoey, P.; Niamsup, H.; Lumyong, S.; Suzuki, T.; Katayama, T.; Chairote, G. Comparison of gamma-aminobutyric acid production in Thai rice grains. *World Journal of Microbiology and Biotechnology* **2010**, *26*, 257-263.
89. Jantasee, A.; Thumanu, K.; Muangsan, N.; Leeanansaksiri, W.; Maensiri, D. Fourier Transform Infrared Spectroscopy for Antioxidant Capacity Determination in Colored Glutinous Rice. *Food Analytical Methods* **2014**, *7*, 389-399.
90. Jiamyangyuen, S.; Nuengchamnong, N.; Ngamdee, P. Bioactivity and chemical components of Thai rice in five stages of grain development. *Journal of Cereal Science* **2017**, *74*, 136-144.
91. Jom, K.N.; Lorjaroenphon, Y.; Udompijitkul, P. Differentiation of four varieties of germinating Thai colored indica rice (Oryza sativa L.) by metabolite profiling. *Food Science and Technology Research* **2016**, *22*, 65-73.
92. Kapcum, N.; Uriyapongson, J.; Alli, I.; Phimphilai, S. Anthocyanins, phenolic compounds and antioxidant activities in colored corn cob and colored rice bran. *International Food Research Journal* **2016**, *23*, 2347-2356.
93. Kittibunchakul, S.; Thiyajai, P.; Suttisansanee, U.; Santivarangkna, C. Determination of GABA content in Thai brown rice by an optimized enzyme-based method. *Chiang Mai Journal of Science* **2017**, *44*, 132-143.
94. Lapmak, K.; Lumyong, S.; Thongkuntha, S.; Wongputtisin, P.; Sardsud, U. L-Asparaginase production by Bipolaris sp. BR438 isolated from brown rice in Thailand. *Chiang Mai Journal of Science* **2010**, *37*, 160-164.
95. Lim, C.W.; Yoshinari, T.; Layne, J.; Chan, S.H. Multi-mycotoxin screening reveals separate occurrence of aflatoxins and ochratoxin A in Asian rice. *Journal of Agricultural and Food Chemistry* **2015**, *63*, 3104-3113.
96. Maisont, S.; Narkrugsa, W. Effects of some physicochemical properties of paddy rice varieties on puffing qualities by microwave "original". *Kasetsart Journal - Natural Science* **2009**, *43*, 566-575.
97. Moongngarm, A. Chemical compositions and resistant starch content in starchy foods. *American Journal of Agricultural and Biological Science* **2013**, *8*, 107-113.
98. Muntana, N.; Prasong, S. Study on total phenolic contents and their antioxidant activities of Thai white, red and black rice bran extracts. *Pakistan Journal of Biological Sciences* **2010**, *13*, 170-174.
99. Pataranawat, P.; Parkpian, P.; Polprasert, C.; Delaune, R.D.; Jugsujinda, A. Mercury emission and distribution: Potential environmental risks at a small-scale gold mining operation, Phichit Province, Thailand. *Journal of Environmental Science and Health - Part A Toxic/Hazardous Substances and Environmental Engineering* **2007**, *42*, 1081-1093.
100. Pengkumsri, N.; Chaiyasut, C.; Saenjum, C.; Sirilun, S.; Peerajan, S.; Suwannalert, P.; Sirisattha, S.; Sivamaruthi, B.S. Physicochemical and antioxidative properties of black, brown and red rice varieties of northern Thailand. *Food Science and Technology* **2015**, *35*, 331-338.
101. Phattayakorn, K.; Pajanyor, P.; Wongtecha, S.; Prommakool, A.; Saveboworn, W. Effect of germination on total phenolic content and antioxidant properties of 'Hang' rice. *International Food Research Journal* **2016**, *23*, 406-409.
102. Pinkaew, H.; Thongngam, M.; Wang, Y.J.; Naivikul, O. Isolated rice starch fine structures and pasting properties changes during pre-germination of three Thai paddy (Oryza sativa L.) cultivars. *Journal of Cereal Science* **2016**, *70*, 116-122.
103. Pintasen, S.; Prom-u-Thai, C.; Jamjod, S.; Yimyam, N.; Rerkasem, B. Variation of grain iron content in a local upland rice germplasm from the village of Huai Tee Cha in northern Thailand. *Euphytica* **2007**, *158*, 27-34.
104. Pitt, J.I.; Hocking, A.D.; Bhudhasamai, K.; Miscamble, B.F.; Wheeler, K.A.; Tanboon-Ek, P. The normal mycoflora of commodities from Thailand. 2. Beans, rice, small grains and other commodities. *International Journal of Food Microbiology* **1994**, *23*, 35-53.

105. Pramai, P.; Jiamyangyuen, S. Chemometric classification of pigmented rice varieties based on antioxidative properties in relation to color. *Songklanakarin Journal of Science and Technology* **2016**, *38*, 463-472.
106. Prom-u-thai, C.; Huang, L.; Rerkasem, B.; Thomson, G.; Kuo, J.; Saunders, M.; Dell, B. Distribution of protein bodies and phytate-rich inclusions in grain tissues of low and high iron rice genotypes. *Cereal Chemistry* **2008**, *85*, 257-265.
107. Puvipirom, J.; Chaiseri, S. Contribution of roasted grains and seeds in aroma of oleang (Thai coffee drink). *International Food Research Journal* **2012**, *19*, 583-588.
108. Puwastien, P.; Judprasong, K.; Pinprapai, N. Development of rice reference material and its use for evaluation of analytical performance of food analysis laboratories. *Journal of Food Composition and Analysis* **2009**, *22*, 453-462.
109. Ranmeechai, N.; Photchanachai, S. Effect of modified atmosphere packaging on the quality of germinated parboiled brown rice. *Food Science and Biotechnology* **2017**, *26*, 303-310.
110. Rattanachitthawat, S.; Suwannalert, P.; Riengrojpitak, S.; Chaiyasut, C.; Pantuwatana, S. Phenolic content and antioxidant activities in red unpolished Thai rice prevents oxidative stress in rats. *Journal of Medicinal Plants Research* **2010**, *4*, 796-801.
111. Rojsuntornkitti, K.; Jittrepotch, N.; Kongbangkerd, T.; Kraboun, K. Substitution of nitrite by Chinese red broken rice powder in Thai traditional fermented pork sausage (Nham). *International Food Research Journal* **2010**, *17*, 153-161.
112. Saenchai, C.; Prom-u-thai, C.; Jamjod, S.; Dell, B.; Rerkasem, B. Genotypic variation in milling depression of iron and zinc concentration in rice grain. *Plant and Soil* **2012**, *361*, 271-278.
113. Singhato, A.; Banjong, O.; Charoonruk, G. Effectiveness and acceptance of the developed educational media on the application of a Thai ethnic snack, Thong Pub, with calcium fortification. *Journal of Ethnic Foods* **2017**, *4*, 58-63.
114. Srinuttrakul, W.; Busamongkol, A. In *Elemental analysis of brown rice by inductively coupled plasma atomic emission spectrometry and instrumental neutron activation analysis*, Energy Procedia, 2014; pp 85-91.
115. Sripum, C.; Kukreja, R.K.; Charoenkiatkul, S.; Kriengsinyos, W.; Suttisansanee, U. The effect of storage conditions on antioxidant activities and total phenolic contents of parboiled germinated brown rice (Khao Dok Mali 105). *International Food Research Journal* **2016**, *23*, 1827-1831.
116. Sripum, C.; Kukreja, R.K.; Charoenkiatkul, S.; Kriengsinyos, W.; Suttisansanee, U. The effect of extraction conditions on antioxidant activities and total phenolic contents of different processed Thai Jasmine rice. *International Food Research Journal* **2017**, *24*, 1644-1650.
117. Srisawa, U.; Panunt, W.; Kaende, N.; Tanuchi, S.; Ithara, A.; Lerdvuthisopo, N.; Hansaku, P. Determination of phenolic compounds, flavonoids, and antioxidant activities in water extracts of Thai red and white rice cultivars. *Journal of the Medical Association of Thailand* **2010**, *93*, S83-S91.
118. Surarit, W.; Jansom, C.; Lerdvuthisopon, N.; Kongkham, S.; Hansakul, P. Evaluation of antioxidant activities and phenolic subtype contents of ethanolic bran extracts of Thai pigmented rice varieties through chemical and cellular assays. *International Journal of Food Science and Technology* **2015**, *50*, 990-998.
119. Tananuwong, K.; Lertsiri, S. Changes in volatile aroma compounds of organic fragrant rice during storage under different conditions. *Journal of the Science of Food and Agriculture* **2010**, *90*, 1590-1596.
120. Tansakul, N.; Limsuwan, S.; Trongvanichnam, K. Fumonisin monitoring in Thai red cargo rice by reversed-phase high-performance liquid chromatography with electrospray ionization ion trap mass spectrometry. *International Food Research Journal* **2012**, *19*, 1561-1566.
121. Thitipramote, N.; Pradmeeteekul, P.; Nimkamnerd, J.; Chaiwut, P.; Pintathong, P.; Thitilerdecha, N. Bioactive compounds and antioxidant activities of red (Brown Red Jasmine)

and black (Kam Leum Pua) native pigmented rice. *International Food Research Journal* **2016**, *23*, 410-414.

122. Vatanasuchart, N.; Niyomwit, B.; Wongkrajang, K. Resistant starch contents and the in vitro starch digestibility of thai starchy foods. *Kasetsart Journal - Natural Science* **2009**, *43*, 178-186.

123. Vichit, W.; Saewan, N. Antioxidant activities and cytotoxicity of thai pigmented rice. *International Journal of Pharmacy and Pharmaceutical Sciences* **2015**, *7*, 329-334.

124. Vittayaporn, V.; Chompreeda, P.; Haruthaithanasan, V.; Rimkeeree, H. Preference mapping of Thai consumers for commercial green tea with roasted brown rice. *Kasetsart Journal - Natural Science* **2010**, *44*, 652-663.

125. Yodmanee, S.; Karrila, T.T.; Pakdeechanuan, P. Physical, chemical and antioxidant properties of pigmented rice grown in Southern Thailand. *International Food Research Journal* **2011**, *18*.

126. Yongsmith, B.; Thongpradis, P.; Klinsupa, W.; Chantrapornchai, W.; Haruthaithanasan, V. Fermentation and quality of yellow pigments from golden brown rice solid culture by a selected Monascus mutant. *Applied Microbiology and Biotechnology* **2013**, *97*, 8895-8902.

127. Aekplakorn, W.; Satheannoppakao, W.; Putwatana, P.; Taneepanichskul, S.; Kessomboon, P.; Chongsuvivatwong, V.; Chariyalertsak, S. Dietary Pattern and Metabolic Syndrome in Thai Adults. *Journal of Nutrition and Metabolism* **2015**, *2015*.

128. Cakmak, I.; Prom-u-thai, C.; Guilherme, L.R.G.; Rashid, A.; Hora, K.H.; Yazici, A.; Savasli, E.; Kalayci, M.; Tutus, Y.; Phuphong, P*., et al.* Iodine biofortification of wheat, rice and maize through fertilizer strategy. *Plant and Soil* **2017**, 1-17.

129. Nakamura, S.; Visarathanonth, P.; Kengkarnpanich, R.; Uraichuen, J.; Konishi, K. Cleaning reduces grain losses of stored rice. *Japan Agricultural Research Quarterly* **2008**, *42*, 35-40.

130. Prathepha, P. Pericarp color and haplotype diversity in weedy rice (O. sativa f. spontanea) from Thailand. *Pakistan Journal of Biological Sciences* **2009**, *12*, 1075-1079.

131. Ranawana, D.V.; Henry, C.J.K.; Lightowler, H.J.; Wang, D. Glycaemic index of some commercially available rice and rice products in Great Britain. *International Journal of Food Sciences and Nutrition* **2009**, *60*, 99-110.

132. Ratanavalachai, T.; Thitiorul, S.; Tanuchit, S.; Jansom, C.; Uttama, S.; Itharat, A. Antigenotoxic activity of Thai Sangyod red rice extracts against a chemotherapeutic agent, doxorubicin, in human lymphocytes by sister chromatid exchange (SCE) assay in vitro. *Journal of the Medical Association of Thailand = Chotmaihet thangphaet* **2012**, *95 Suppl 1*, S109-114.

133. Saman, P.; Fuciños, P.; Vázquez, J.J.; Pandiella, S.S. Fermentability of brown rice and rice bran for growth of human Lactobacillus plantarum NCIMB 8826. *Food Technology and Biotechnology* **2011**, *49*, 128-132.

134. Sangthong, R.; Wichaidit, W.; McNeil, E.; Chongsuvivatwong, V.; Chariyalertsak, S.; Kessomboon, P.; Taneepanichskul, S.; Putwatana, P.; Aekplakorn, W. Health behaviors among short- and long- term ex-smokers: Results from the Thai National Health Examination Survey IV, 2009. *Preventive Medicine* **2012**, *55*, 56-60.

135. Sinthorn, W.; Chatuphonprasert, W.; Chulasiri, M.; Jarukamjorn, K. Thai red rice extract provides liver protection in paracetamol-treated mice by restoring the glutathione system. *Pharmaceutical Biology* **2016**, *54*, 770-779.

136. Son, J.S.; Do, V.B.; Kim, K.O.; Cho, M.S.; Suwonsichon, T.; Valentin, D. Consumers' attitude towards rice cooking processes in Korea, Japan, Thailand and France. *Food Quality and Preference* **2013**, *29*, 65-75.

137. Suwannalert, P.; Rattanachitthawat, S.; Chaiyasut, C.; Riengrojpitak, S. High levels of 25-hydroxyvitamin D <inf>3</inf> [25(OH)D <inf>3</inf>] and α-tocopherol prevent oxidative stress in rats that consume Thai brown rice. *Journal of Medicinal Plants Research* **2010**, *4*, 120-124.

138. Tammasakchai, A.; Reungpatthanaphong, S.; Chaiyasut, C.; Rattanachitthawat, S.; Suwannalert, P. Red strain Oryza sativa-unpolished thai rice prevents oxidative stress and

colorectal aberrant crypt foci formation in Rats. *Asian Pacific Journal of Cancer Prevention* **2012**, *13*, 1929-1933.
139. Teangpook, C.; Paosangtong, U. The production and shelf life of high-iron, pre-cooked rice porridge with ferrous sulphate and other high-iron materials. *Maejo International Journal of Science and Technology* **2011**, *5*, 279-291.
140. Thongoun, P.; Pavadhgul, P.; Bumrungpert, A.; Satitvipawee, P.; Harjani, Y.; Kurilich, A. Effect of oat consumption on lipid profiles in hypercholesterolemic adults. *Journal of the Medical Association of Thailand = Chotmaihet thangphaet* **2013**, *96 Suppl 5*, S25-32.
141. Wunjuntuk, K.; Kettawan, A.; Rungruang, T.; Charoenkiatkul, S. Anti-fibrotic and anti-inflammatory effects of parboiled germinated brown rice (Oryza sativa 'KDML 105') in rats with induced liver fibrosis. *Journal of Functional Foods* **2016**, *26*, 363-372.

Vietnam

142. Phuong, T.D.; Van Chuong, P.; Khiem, D.T.; Kokot, S. Elemental content of Vietnamese rice. Part 1. Sampling, analysis and comparison with previous studies. *Analyst* **1999**, *124*, 553-560.
143. Kokot, S.; Phuong, T.D. Elemental content of Vietnamese rice. Part 2. Multivariate data analysis. *Analyst* **1999**, *124*, 561-569.
144. Rahman, M.A.; Rahman, M.M.; Reichman, S.M.; Lim, R.P.; Naidu, R. Heavy metals in Australian grown and imported rice and vegetables on sale in Australia: Health hazard. *Ecotoxicology and Environmental Safety* **2014**, *100*, 53-60.
145. Wu, T.-T.; Charles, A.L.; Huang, T.-C. Determination of the contents of the main biochemical compounds of Adlay (Coxi lachrymal-jobi). *Food chemistry* **2007**, *104*, 1509-1515.
146. Bui, T.N.; Le Hop, T.; Nguyen, D.H.; Tran, Q.B.; Nguyen, T.L.; Le, D.T.; Nguyen, D.V.A.; Vu, A.L.; Aoto, H.; Okuhara, Y.*, et al.* Pre-germinated brown rice reduced both blood glucose concentration and body weight in vietnamese women with impaired glucose tolerance. *Journal of Nutritional Science and Vitaminology* **2014**, *60*, 183-187.
147. Wopereis-Pura, M.; Watanabe, H.; Moreira, J.; Wopereis, M. Effect of late nitrogen application on rice yield, grain quality and profitability in the Senegal River valley. *European Journal of Agronomy* **2002**, *17*, 191-198.
